# CLUSTOM: A Novel Method for Clustering 16S rRNA Next Generation Sequences by Overlap Minimization

**DOI:** 10.1371/journal.pone.0062623

**Published:** 2013-05-01

**Authors:** Kyuin Hwang, Jeongsu Oh, Tae-Kyung Kim, Byung Kwon Kim, Dong Su Yu, Bo Kyeng Hou, Gustavo Caetano-Anollés, Soon Gyu Hong, Kyung Mo Kim

**Affiliations:** 1 Biological Resource Center, Korea Research Institute of Bioscience and Biotechnology, Daejeon, Korea; 2 Department of Bioinformatics, University of Science and Technology, Daejeon, Korea; 3 Korean Bioinformation Center, Korea Research Institute of Bioscience and Biotechnology, Daejeon, Korea; 4 Department of Systems Biology, Yonsei University, Seoul, Korea; 5 Evolutionary Bioinformatics Laboratory, Department of Crop Sciences, University of Illinois, Urbana, Illinois, United States of America; 6 Division of Polar Life Sciences, Korea Polar Research Institute, Incheon, Korea; University of Houston, United States of America

## Abstract

The recent nucleic acid sequencing revolution driven by shotgun and high-throughput technologies has led to a rapid increase in the number of sequences for microbial communities. The availability of 16S ribosomal RNA (rRNA) gene sequences from a multitude of natural environments now offers a unique opportunity to study microbial diversity and community structure. The large volume of sequencing data however makes it time consuming to assign individual sequences to phylotypes by searching them against public databases. Since ribosomal sequences have diverged across prokaryotic species, they can be grouped into clusters that represent operational taxonomic units. However, available clustering programs suffer from overlap of sequence spaces in adjacent clusters. In natural environments, gene sequences are homogenous within species but divergent between species. This evolutionary constraint results in an uneven distribution of genetic distances of genes in sequence space. To cluster 16S rRNA sequences more accurately, it is therefore essential to select core sequences that are located at the centers of the distributions represented by the genetic distance of sequences in taxonomic units. Based on this idea, we here describe a novel sequence clustering algorithm named CLUSTOM that minimizes the overlaps between adjacent clusters. The performance of this algorithm was evaluated in a comparative exercise with existing programs, using the reference sequences of the SILVA database as well as published pyrosequencing datasets. The test revealed that our algorithm achieves higher accuracy than ESPRIT-Tree and mothur, few of the best clustering algorithms. Results indicate that the concept of an uneven distribution of sequence distances can effectively and successfully cluster 16S rRNA gene sequences. The algorithm of CLUSTOM has been implemented both as a web and as a standalone command line application, which are available at http://clustom.kribb.re.kr.

## Introduction

Microorganisms play critical roles in regulating the biogeochemistry of our planet. Microbial communities largely influence the relationships of biotic and abiotic environments. Assessing microbial diversity (“Who is out there?”) is however the first step in understanding the role of microbes in biogeochemical evolution. Since a universal taxonomic structure of life based on ribosomal RNA (rRNA) was established in mid-1980s [1], ribosomal genes have been used as ‘gold standard’ to identity species and build higher-level taxonomies. Based on this taxonomical structure, the sequencing of 16S rRNA genes (16S) derived from environmental samples led to the discovery of unprecedented diversity of both cultured and uncultured microbes [2]. In addition, next generation sequencing (NGS) technologies such as pyrosequencing and Illumina are producing high-volume information at DNA level, facilitating the unprecedented detection of new phylotypes. There are however important technical limitations that need to be overcome. Although shotgun-based metagenomic data is becoming increasingly available, surveying extensively the genetic diversity of a specific gene in most microbial communities is not sufficient [3]. For this reason, there is still great demand for 16S sequences of environmental samples. Similarly, the analysis of 16S data generated from high-throughput amplicon-based experiments represents a bioinformatics challenge that requires accurate and efficient handling of data.

The analysis of the structure of a microbial community starts with the estimation of α-diversity parameters. α-diversity is calculated by considering how rich and evenly distributed are microbial taxa across taxonomic groups. These estimates can be determined by comparing gene-targeted sequences (e.g., 16S/18S, *rpoB*, etc) against reference databases with taxonomic information. However, the usefulness of this taxonomy-dependent approach largely depends on the quality and quantity of sequences recorded in the reference databases. Since deposited sequences so far are highly limited and only represent a minority of the extant microbial world, this approach introduces bias in microbial diversity estimates, mostly missing uncultured microbes. On the other hand, a taxonomy-independent approach conducts *de novo* comparison of sequences, clusters sequences into operational taxonomic units (OTUs) under given sequence distance thresholds, and usually discovers a larger number of OTUs than the taxonomy-dependent approach. Furthermore, the use of sequences that represent OTUs considerably reduces computational complexity in analyzing massive 16S data. The algorithms used by this approach are commonly classified into greedy heuristic clustering (e.g., CD-hit [4], UCLUST [5]) and hierarchical clustering algorithms. The latter is further categorized into single- (SL), complete- (CL), and average-linkage (AL) clustering methods that are partially or fully supported by a couple of programs (e.g., DOTUR [6], ESPRIT [7], ESPRIT-Tree [8], mothur [9] and TBC [10]). While the greedy heuristic clustering is computationally more efficient, its accuracy is considerably lower than that of hierarchical clustering algorithms [8]. In fact, the accuracy of clustering algorithms largely depends on the accuracy of the genetic distances of sequence pairs. The distances are more accurately calculated from pairwise sequence alignment (PSA) than from multiple sequence alignment (MSA) since the alignment of any two sequences in a MSA is affected by the constraint of preserving positional homology across multiple aligned sequences [11], [12]. While the Needleman–Wunsch (NW) algorithm exhibits the highest computational complexity, it exhaustively compares two sequences and provides an optimal PSA. Therefore, the use of NW followed by clustering is the ideal clustering approach to identify OTUs accurately. This can be accomplished using NW-DOTUR-AL or NW-mothur-AL.

Promiscuous genetic exchanges may occur between bacterial species as a result of processes of interspecific recombination. Despite this promiscuity, genetic divergence between species remains evident. This divergence is revealed by the highly resolved relationships of bacterial lineages and suggests speciation results from genetic discontinuity between closely related populations [13]. A recent study that examined the nature of bacterial speciation under neutral models (e.g., Fisher-Wright model) shows that recombination rate is negatively correlated with the degree of species divergence [14]. This recombinational incompatibility will likely make bacterial isolates that are genetically similar more homogeneous and will eventually lead to speciation [15]. A recent study for a marine microbial community also supported the concept that gene sequences can be homogenous within species but can diverge between species in the natural environment [16]. In these cases, interspecies recombination between rRNA genes is unlikely, although few exceptions have been observed in prokaryotic lineages (e.g., extreme halophilies [17]). These results suggest that both 16S genotype clustering within species and genetic isolation between species can be unevenly present in sequence space when coordinates are expressed in units of 16S sequence divergence. Consequently, the selection of core sequences that are located at the centers of the clusters in the distribution would result in the minimization of overlaps between adjacent clusters in sequence space. Based on this biological premise, we here describe a novel clustering algorithm. This algorithm is probably the first to consider the nature of prokaryotic speciation in clustering 16S rRNA sequences.

Determining which clustering method is the best remains controversial despite recent benchmark studies that proposed AL-based hierarchical clustering methods to be the most accurate [12], [18], [19]. We here compare the performance of our algorithm relative to that of ESPRIT-Tree, NW-DOTUR-AL and NW-mothur-AL, all of which use the average clustering algorithms. In order to evaluate how differently alignment methods affect the clustering results between the programs, we additionally tested NAST-mothur-AL, which indicates aligning of sequences by comparing them with a reference 16S sequence database using NAST program followed by clustering sequences using mothur with the average linkage algorithm. The classical MSA that conducts de novo multiple sequence alignment without the aid of any reference database was not evaluated since it suffers from the problem of positional homology as mentioned above [12]. UCLUST has been used widely as part of QIIME package. However, this program was not comparatively evaluated in our analysis since its algorithm is originated from not AL-based hierarchical clustering but greedy-based heuristic clustering [5]. A comparative analysis of 16S data from pyrosequencing and the SILVA database showed that the performance of our algorithm was comparable to NW-DOTUR-AL but outperformed any other programs in terms of accuracy. Because our algorithm utilizes more computational resources in comparison to ESPRIT-Tree and NAST-mothur-AL, we therefore developed and made available a parallel processing standalone application and web system named CLUSTOM that can analyze large-scale sequence data efficiently and in a user-friendly manner.

## Materials and Methods

### Overview of the Algorithm

**Figure 1:**
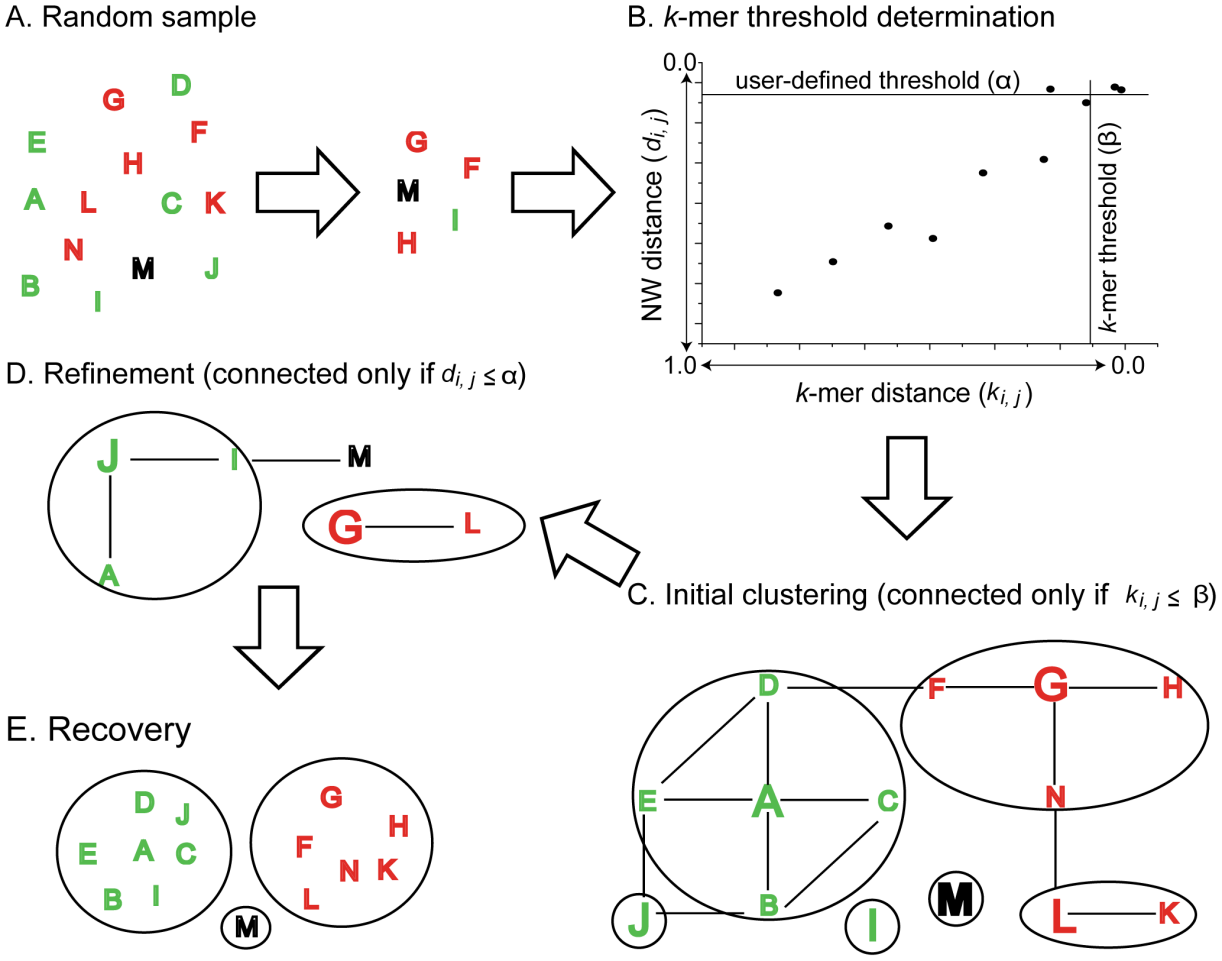
Schematic representation of the algorithmic workflow. The diagram summarizes the flow of the CLUSTOM algorithm. There are five main steps that are processed sequentially. (A) *Random sample*: Here we assume that the input sequences (individual letters) are clustered into three OTUs, labeled in green, red, and black. A sequence subset is randomly extracted from these sequences. (B) *k-mer threshold determination*: Individual dots indicate pairs of the randomly sampled sequences. Distances of *k*-mer and Needleman-Wunsch (NW) between sequences *i* and *j* are denoted by *k_i,j_* and *d_i,j_*, respectively. The user-defined distance threshold and its corresponding *k*-mer threshold are respectively denoted by α and β, respectively. (C) *Initial clustering*: If *k*-mer distances of any two of the input sequences are smaller than the *k*-mer threshold (β), they are connected in a network. The larger letters in bold indicate the seed sequences of initial clusters that are bound by circles. (D) *Refinement*: Seed sequences with NW distances smaller than the user-defined threshold (α) are used to construct a refined network following the procedures in (C). The larger letters in bold indicate the refined seed sequences. (E) *Recovery*: Each of the final clusters (circles) consists of the refined seed, its neighbors, and sequences that are directly connected to the refined seed or the neighbors in the *initial clustering* step.

The algorithm of CLUSTOM clusters high-throughput 16S sequences using user-defined thresholds. It consists of the following three main steps: (i) *k-mer threshold determination*: A subset of sequences is randomly sampled from 16S input sequences (Figure 1A). For all possible sequence pairs that are sampled, the algorithm calculates *k*-mer distances and dissimilarity of PSA using the NW algorithm (NW distance). Regarding the correlation between the two distance variables, the sequence pairs are categorized into true positives (TP), false positives (FP), false negatives (FN), and true negatives (TN). Here, the user-defined and the corresponding *k*-mer thresholds are denoted by α and β, respectively. Distances of *k*-mer and NW between a sequence pair (*i* and *j*) are indicated by *k_i,j_* and *d_i,j_*, respectively. The four categories are now defined as follows: TP = sequence pairs with distances *d_i,j_*≤α and *k_i,j_*≤β; FP = sequence pairs with distances *d_i,j_*>α and *k_i,j_*≤β; FN = sequence pairs with distances *d_i,j_*≤α and *k_i,j_*>β; and TN = sequence pairs with distances *d_i,j_*>α and *k_i,j_*>β (Figure 1B). The CLUSTOM algorithm searches for the *k*-mer distance value at which FP becomes zero. This point is regarded as the *k*-mer threshold (β) that corresponds to the user-defined threshold (α; Figure 1B); (ii) *Initial clustering*: CLUSTOM then calculates *k*-mer distances of every pair of input sequences, selects the sequence pairs that satisfy the *k*-mer threshold determined above (*k_i,j_*≤β; Figure 1B) and constructs a network with the sequence pairs that were chosen (Figure 1C). The algorithm then searches for the most connected node in the network that most likely represents a core sequence that is located at the centers of the clusters in sequence space and regards it as a seed sequence (the letter ‘A’ in Figure1C). We note that since a virtual node, which is not present by any sequences of the cluster, will sometimes represent the core sequence that is located at the center of a cluster, it is impossible to select a core sequence for every cluster. This is why we regarded the most connected node as the cluster center. However, the close relatedness between the most connected nodes and the centers of clusters is expected, given the higher accuracy of our algorithm in comparison to other clustering programs (see Results). The first initial cluster is then determined by the seed and sequences directly connected to the seed (the letters ‘B’, ‘C’, ‘D’, and ‘E’ in Figure 1C). Then, the algorithm excludes the sequences of the cluster from the existing network, searches for the next most connected sequence in the new network that consists of the remaining sequences, determines the next seed (the letter ‘G’ in Figure 1C), and clusters its neighbors (the letters ‘F’, ‘H’, and ‘N’). This sequential process is iteratively repeated until only singletons (one sequence per cluster) remain; and (iii) *Refinement*: Although *k*-mer distance is strongly correlated with NW distance [4], [5], [7], [8], the relationship between the two variables is not completely linear. Therefore, sequence pairs with NW distances less than α that has *k*-mer distances more than β can occur. Since they are regarded as false negatives in the *k-mer threshold determination* step, these sequence pairs are not present in the networks of the *initial clustering* step and remain as singletons. In order to include these false negative singletons in the analysis a refinement process is conducted by the algorithm. NW is first completed for every pair of the seed sequences in the *initial clustering* step (letters ‘A’, ‘G’, ‘L’ in Figure 1C) as well as the singletons (letters ‘I’, ‘J’, ‘M’ in Figure 1C). From the sequence pairs that satisfy the user-defined threshold (*d_i,j_*≤α), a network is constructed (Figure 1D). The most connected node becomes a refined seed. Then, the final cluster consists of the refined seed (the letter ‘J’ in Figure1D), its neighbors (the letters ‘A’ and ‘I’ in Figure 1D), and sequences that are directly connected to the refined seed or the neighbors in the *initial clustering* step (the letters ‘B’ and ‘E’ to ‘J’; ‘B’, ‘C’, ‘D’, and ‘E’ to ‘A’; no node to ‘I’; see Figure 1C–E). The determination of the next clusters is conducted in the same way as in the *initial clustering* step. Below, we describe detailed methodologies that were used in the development of the CLUSTOM algorithm.

### Gap Treatment and NW Distance

The precise calculation of PSA is important for accurate clustering. We thus used NW to obtain optimal PSAs for given sequence pairs. Each of the PSAs was edited by removing end gaps as well as internal gaps before calculating the NW distance. In fact, there is no general consensus of how internal gaps should be treated in this calculation. Three options can be consider: (i) ignoring all gaps; (ii) regarding consecutive gaps as a single event [7], [8]; and (iii) allowing all gaps as they are. Due to the hyper-variable regions of 16S sequences, the first two options have been widely used. In fact, they generate nearly identical sequence distances [11]. However, most of 16 rRNA surveys conducted in phylogenetic and ecological contexts have used substitution models that regard any indels as missing data [10], [20], [21]. Furthermore, option (ii) cannot resolve indels from homopolymeric errors in the analysis of pyrosequences. We therefore used option (i) to calculate the NW distance
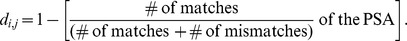



On the other hand, *k*-mer distance for a sequence pair was calculated by the formula
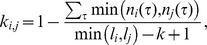
where 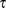 indicates a *k*-mer. 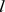 is the length of sequences (*i* or *j*), and 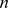 is the number of occurrences of 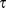 in the sequences.

### Data Preparation for Benchmark Studies

To compare the clustering accuracy of CLUSTOM, NW-DOTUR-AL, ESPRIT-Tree, NAST-mothur-AL and NW-mothur-AL, we prepared test datasets from three different sources. We first retrieved bacterial 16S sequences from the database of the SILVA database, release 108, which are reliably curated by considering alignment quality and phylogenetic relationships. The sequences that had duplicated accession numbers or were shorter than 1,200 nt in length were removed. Referring to the LPSN database (http://www.bacterio.cict.fr/ac.html), we extracted the sequences to which valid scientific names are assigned. Consequently, only 27,213 16S sequences (1451 bp on average) that are referred to as 16S–SILVA in this study were prepared. The second dataset that is referred to as 454–HMP was curated from 16S sequences of microbial communities that were isolated from various human body sites using the Roche-454 FLX Titanium platform (NCBI accession: SRP002395). This data was retrieved from the data archive of the Human Microbiome Project (http://hmpdacc.org/HM16STR). The dataset contains over 7×10^7^ reads that were already trimmed and processed. The third dataset that are referred to as 454–SPONGE was prepared from public 16S pyrosequencing sequences of the V1–3 region [22]. The 454–SPONGE dataset consists of complex, simple, and intermediate bacterial communities that are associated with marine sponges *Raspailia ramose* (24,433 reads) and *Stelligera stuposa* (26,918 reads), and seawater (18,271 reads) collected from the sponge-sampling site, respectively. Since the 454–SPONGE dataset was not fully processed, we removed sequencing errors using AmpliconNoise [23] and trimmed tags (barcodes, linkers and primers) using an in-house developed script. As a result, three processed datasets of *R.* *ramose* (12,898 reads, 456 bp on average), *S. stuposa* (10,898 reads, 471 bp on average), and seawater (9,944 reads, 397 bp on average) were prepared.

### Visualization of 16S NW Distances

The linear dependence between *k*-mer and NW distances was examined by calculating the Pearson product-moment correlation using an in-house developed script. To visualize the extent of the closeness between 16S sequences, NW distances of the sequence pairs were calculated and plotted in three-dimensional space using Principal Component Analysis (PCA) implemented in R ver. 2.15.1.

### Taxonomy-based Evaluation

To evaluate the clustering accuracy of CLUSTOM and compare it to that of NW-DOTUR-AL, ESPRIT-Tree, NAST-mothur-AL and NW-mothur-AL, we used the 27,213 16S–SILVA sequences with taxonomic information. We assigned sequences to OTUs with the five programs at species and genus taxonomical levels with 3% and 5% conventional thresholds, respectively [7], [24]. We then counted how many sequences of individual taxa, each of which indicates a species or a genus, were assigned to the OTUs. For every taxon, we identified the OTU that had the largest number of taxon sequences. This OTU was regarded as the representative cluster of the taxon. If multiple taxa had the same maximum abundance in a cluster, they were regarded as a composite taxon by sharing the single OTU as their representative. On the other hand, taxa that were equally represented by multiple OTUs were excluded in this calculation to avoid statistical bias. For each of the taxa, we calculated TP, FP, FN, and TN as follows: (i) TP = the number of sequences of the target taxon in its representative OTU; (ii) FP = the number of sequences of the remaining taxa in the representative OTU; (iii) FN = the number of sequences of the target taxon in the other OTUs; and (vi) TN = the number of sequences of the remaining taxa in the other OTUs. We illustrate the procedure with a simple toy example below. Let us assume that a dataset consists of 15, 8, and 17 sequences for species A, B, and C, respectively. Then, we assume that the 40 sequences were clustered into three OTUs as follows: OTU1 (A, B, C) = (10, 5, 2), OTU2 (A, B, C) = (2, 1, 10), and OTU3 (A, B, C) = (3, 2, 5). In this case, OTU1 is the representative cluster of species A and B since the numbers of sequences of A and B present in the OTU1 are larger than those in the OTU2 and OTU3. Therefore, species A and B are regarded as a composite taxon. The values of TP, FP, FN, and TN for the composite taxon are calculated as follows: TP = c1(A)+c1(B) = 15, FP = c1(C) = 2, FN = c2(A)+c2(B)+c3(A)+c3(B) = 8, and TN = c2(C)+c3(C) = 15, where the letter c indicates a cluster. We calculated in this way the four values for every taxon determined. We then computed precision TP/(TP+FP) and recall TP/(TP+FN) for every taxon. Here, precision means the fraction of correctly assigned sequences out of all the sequences in the OTU of a target taxon, whereas recall is defined as the fraction of correctly assigned sequences out of all the sequences in all the OTUs. The harmonic means of the precision and recall averaged over all taxa were calculated using the formula of F-measure II (*F_2_*) [25].

### Distance-based Evaluation

Although the 16S–SILVA dataset was carefully curated, two types of taxonomic assignment errors can occur: (i) identical sequences with different species labels; and (ii) highly diverged sequences with the same species label. These problems can bias the taxonomy-based accuracy of the five programs that were evaluated above. Therefore, it is necessary to evaluate the program accuracy in the taxonomy-independent viewpoint. Furthermore, most of 16S sequences recently produced by NGS are shorter in length than those of the 16S–SILVA dataset, which have been mostly sequenced by the Sanger method. In this regard, we evaluated clustering accuracy in the most realistic condition, by running the five programs using the 454–SPONGE datasets. We examined how well the individual programs cluster sequences for given distance thresholds since no taxonomy information is available for these 454 datasets. The concept of false conjunction and false disjunction was applied [10]. While the former means wrong assignments of sequences within an OTU, the latter indicates incorrect separation of sequences between OTUs. Since the formulas described by [10] were developed to evaluate the clustering results of the CL methods, they are not appropriate for the AL-based approach used by the five programs we are evaluating. We thus modified the equations by determining a core sequence that has the minimum average distance with the other sequences for every OTU and calculated the rates of false conjunction and disjunction using the following re-formulated equations, respectively:
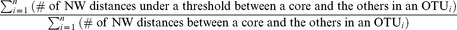
and



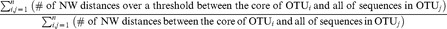



### CLUSTOM Implementation

The CLUSTOM algorithm was implemented as a C program. In order to quickly complete NW calculation that is computationally heavy, this algorithm supports parallel computation using multiple CPU cores. CLUSTOM can calculate clustering results for distance thresholds from 3% to 10% in a single run. Regardless of user-defined thresholds, the first two steps are conducted only once using the threshold of 3% (Figure 1). The sizes of the random sample (Figure 1A) and *k*-mer are the default setting for the *k-mer cutoff determination* step but the former can be adjusted by the user (for detailed simulation results, see Results). CLUSTOM then accomplishes the *refinement* step at every one of the user-defined thresholds with the increment of 0.01. The standalone compilable source code and user guide as well as the parallel processing web application are available at http://clustom.kribb.re.kr. The web server that is equipped with four sixteen-core 2.4 GHz CPU cores and 192 GB memory assigns 20 CPU cores to individual queries and can process three different jobs simultaneously. This web server allows a user to upload up to 300 K sequences. Queries pended are automatically run by an internal job scheduler. CLUSTOM accepts a FASTA file of 16S sequences as input, validates the input format internally, clusters them to OTUs, and outputs a couple of files that represent: (i) sequences per OTU; (ii) the representative sequences of OTUs; and (iii) the number of sequences per OTU.

### Running Time

We measured the running time of CLUSTOM using the computer cluster described above. The sequence datasets (479 to 487 bp on average) of 10 K, 20 K, 50 K, 100 K, and 200 K sizes that were randomly sampled from the V3–5 region of the 454–HMP were analyzed. Depending on the size of the datasets, CLUSTOM was selectively run using one, four, eight, and 20 CPU cores. Since CLUSTOM can conduct clustering consecutively for multiple discrete distance thresholds, we only used the threshold of 3% to measure the running time.

## Results

### Uneven Distribution of 16S Sequences

**Figure 2:**
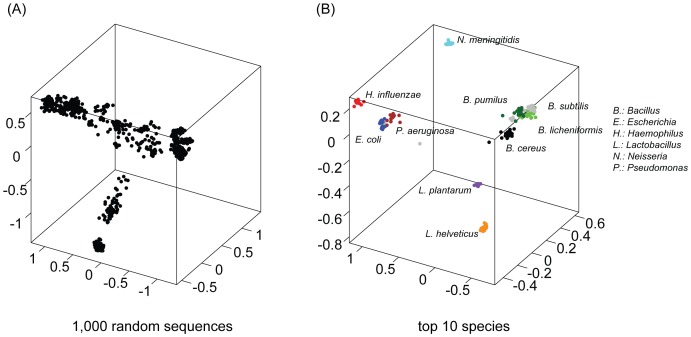
Genetic cartography of 16S sequence distances. Plots show the uneven distribution of 16S sequences visualized in sequence space. Coordinates are expressed in units of NW distances of sequence pairs. (A) PCA analysis of 1,000 sequences randomly sampled from the 16S–SILVA dataset showing the relationships of NW distances. (B) PCA analysis of the sequences of ten species that were the most abundantly present in the set of the 1,000 sequences displayed in (A).

To justify our algorithmic premise, we examined whether 16S sequences are clustered well within species and are separated from other species. The relationships between sequences were measured with NW distances between the possible pairs of 1,000 sequences that were randomly sampled from the 16S–SILVA dataset. The genetic cartography of the sequences was generated using PCA (Figure 2A). The plot shows that the randomly sampled sequences are unevenly distributed along the coordinates of NW distances. We then chose the top ten species that were the most abundantly present in the set of the 1,000 sequences and produced a PCA plot of their sequence relationships (Figure 2B). To evaluate the reliability of species clusters present in the plot, we first calculated genetic distance between the 16S sequences of type strains of every species pair using the ARB package [26]. Some species that include *Bacillus cereus* (in black), *Haemophilus influenzae* (red), *Lactobacillus plantarum* (violet), *L. helveticus* (orange), and *Neisseria meningitidis* (cyan) were distinctly clustered (Figure 2B), whereas the distribution of the other species was overlapped. However, the ARB analysis showed that 16S sequences of type strains of the three *Bacillus* species (*B. licheniformis* [in green], *B. pumilus* [dark green], *B. subtilis* [gray]) are nearly identical, indicating that their overlaps in the PCA plot are expected natural phenomena. While *Escherichia coli* (in blue) and *Pseudomonas aeruginosa* (brown) are phylogenetically distant, the PCA plot shows the close relationships between the two species. However, we observed that this overlap was distinctly resolved by rotating axes (data not shown). Consequently, the PCA patterns congruently describe phylogenetic relationships and shows that sequences of a species are cohesively clustered and distinctly resolved from other species, supporting the reliability of the algorithmic premise of CLUSTOM.

### Determining an Optimal *k*-mer Size

**Figure 3:**
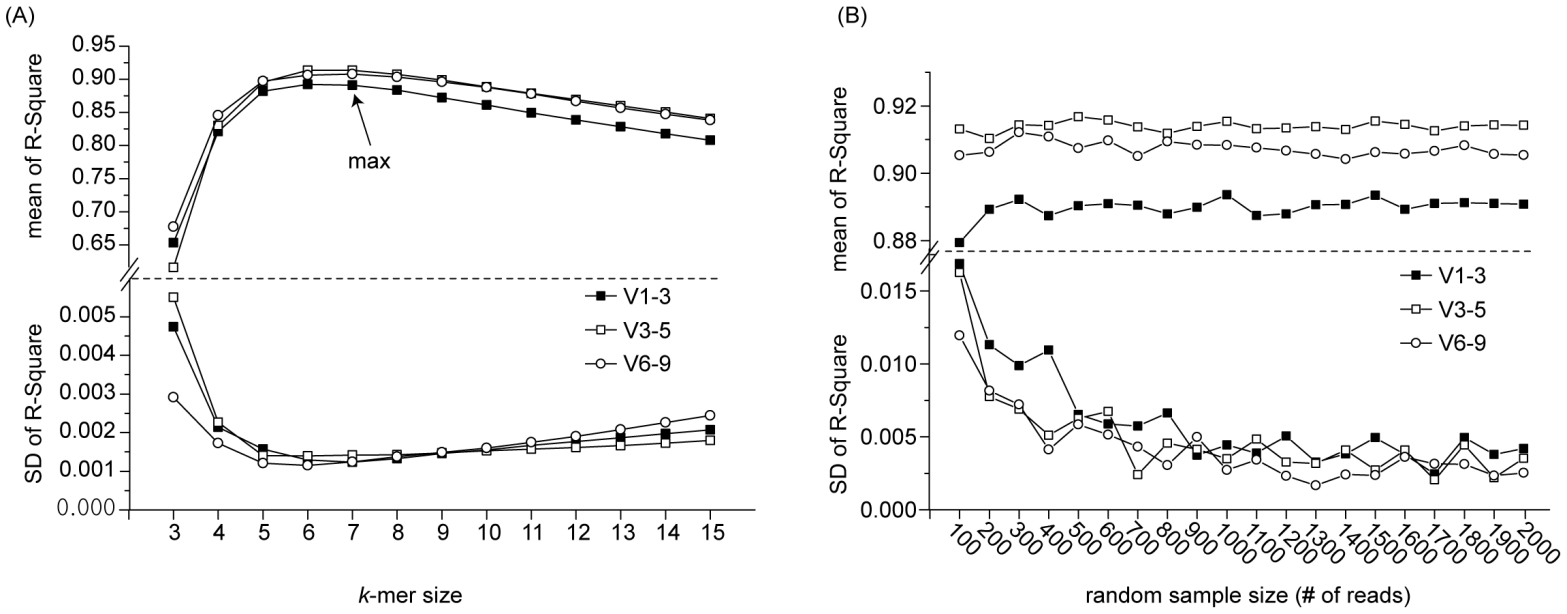
Parameter optimization. We simulated three variable regions of the 454–HMP dataset (V1–3, V3–5, and V6–9) to determine optimal sizes of *k*-mer and random sample. (A) From each region, 10 K reads were randomly sampled 10 times and NW distances calculated. *k*-mer distances between the pairs of the 10 K reads were calculated for each of *k*-mer sizes ranging from 3 to 15. The extent of linear regression between the two distance variables was plotted using the mean (upper *x* axis) and standard deviation (lower *x* axis) of the square of the Person product-moment correlation coefficients (R-Square) at every point of the *k*-mer sizes. (B) For individual 16S regions, we randomly sampled sequences of 100 to 2,000 reads in increments of 100 reads. The *k*-mer and NW distances were calculated for all possible sequence pairs at every point of the random sample sizes. The random sampling followed by the distance calculation was repeated 10 times. For ten random samples per sample size, the mean (upper *x* axis) and standard deviation (lower *x* axis) of R-Square were calculated and displayed.

NGS technologies such as pyrosequencing and Illumina are capable of generating tens of thousands of 16S sequences per microbial community. The number of possible sequence pairs that can be compared is immensely high. For example, analysis of 50,000 sequences (_n_C_2_) represents over a billion pairwise comparisons. Furthermore, the computational complexity of the NW algorithm is close to *O(N^2^)*, where *N* is the length of the larger sequence of the pairwise comparison. It is therefore impossible to calculate NW distances for all possible pairs of the sequences. The *k*-mer distance shows good correlation with the NW distance [4], [5], [7], [8]. The appropriate *k*-mer threshold that corresponds to a given user-defined cutoff is expected to greatly reduce computational complexity. We here conducted sequence simulations to optimize parameters related to the *k*-mer threshold. An optimal *k*-mer size was first determined. We extracted V1–3 (ca. 14 million reads), V3–5 (3 million), and V6–9 (half a million) sequences from the 454–HMP dataset that range from 450 to 500 bp in length. From each region, we randomly sampled 10 K sequences. This sampling was repeated 10 times to reduce statistical bias. Every sequence pair was aligned using the NW algorithm and its distance was calculated by ignoring gaps as described above. In parallel, *k*-mer distances were calculated for each of the *k* sizes that range from 3 to 15. The linear dependence between *k*-mer and NW distances was examined for individual sizes by calculating the square of the Pearson product-moment correlation coefficient, referred to as R-Square, using an in-house developed script. As a result, the mean of R-Square values increases with the increase of *k*-mer sizes and reaches the maximum at *k*-mer sizes of 7 for all the regions (Figure 3A). The correlation between the two distance variables is stronger with larger R-Square values. Consequently, we determined an optimal *k*-mer size of seven nucleotides in length, which is similar to the default used in both the ESPRIT and ESPRIT-Tree programs (*k* = 6). As a result, the optimal size is fixed in CLUSTOM as default for clustering 16S sequences.

### Determining the Minimum Size of a Random Sample

Since sequences generated by the NGS platforms vary between 100 and 650 bp in length, they cannot cover the full-length sequences of the 16S genes. Furthermore, the *k*-mer threshold that corresponds to a user-defined NW cutoff can change depending on which sequence regions are selected. This means that the *k*-mer threshold should not be fixed in a clustering algorithm. Instead, it should be calculated from a given input sequence dataset. The CLUSTOM algorithm thus randomly samples a small number of sequences from a given input data, computes both *k*-mer and NW distances for every sequence pair, and determines the approximate *k*-mer threshold. To minimize computing time in this calculation, we estimated how many sequences must be sampled. From each of the variable regions (V1–3, V3–5, V6–9) extracted from the 454–HMP dataset, we randomly sampled sequences of 100 to 2,000 reads with an increment of 100 reads and calculated both *k*-mer and NW distances for all possible pairs. The random sampling was repeated 10 times for each sample size. The mean and standard deviation of R-Square were obtained for 10 random samples per sample size (Figure 3B). The plot shows that the R-Square values at relatively small sample sizes are highly variable. As the sample sizes increase, the standard deviations become smaller and more stable. At sample size of 1,000, the R-Square values are nearly invariable and their standard deviations are less than 0.005. This value is generally used as stringent cutoff to judge the stationary variation of a given parameter. Consequently, we set the minimal sample size to 1,000 in CLUSTOM as default and consider this value provides a stable correlation between *k*-mer and NW distances stable.

### *k*-mer Threshold and Clustering

CLUSTOM randomly samples sequences with the sample size determined above (Figure 1A) and computes *k-*mer and NW distances between the sequence pairs. In order to determine the *k*-mer threshold that corresponds to the user-defined distance cutoff, the following two criteria can be considered: (i) minimizing the sum of FPs and FNs; and (ii) minimizing FPs. While the numbers of TP and FP are positively correlated, they exhibit a tradeoff relationship with FNs. Consequently, the *k*-mer threshold under the first criterion produces a relatively large number of TPs with the smallest number of FNs and FPs. However, we expect that FPs will be present downstream during clustering analysis. On the other hand, the *k*-mer threshold under the second criterion does not allow any FPs at least in the random sequence sample while it decreases the number of TPs. To our knowledge, there is no effective way to exclude FPs in the *initial clustering* step. On the other hand, the CLUSTOM algorithm can supplement the loss of TPs at the *refinement* step (Figure 1D) without heavy computational complexity. For this reason, we selected the second criterion to determine the optional *k*-mer threshold (β in Figure1B). Although CLUSTOM determines the *k*-mer threshold from a subset of input sequences, it is most likely that the network in the *initial clustering* step consists of sequence pairs that satisfy the user-defined distance threshold. In the *initial clustering* step, CLUSTOM extracts seed sequences that are the most connected nodes of the network. These sequences represent a minority of input sequences (e.g., around 0.1 to 4% in the datasets of this study). Since only the seed sequences are compared with the NW algorithm, the use of *k*-mer thresholds followed by the *refinement* step greatly reduces computational complexity in comparison to exhaustive NW comparison between all possible sequence pairs.

### Taxonomy-based Reliability Test

**Figure 4:**
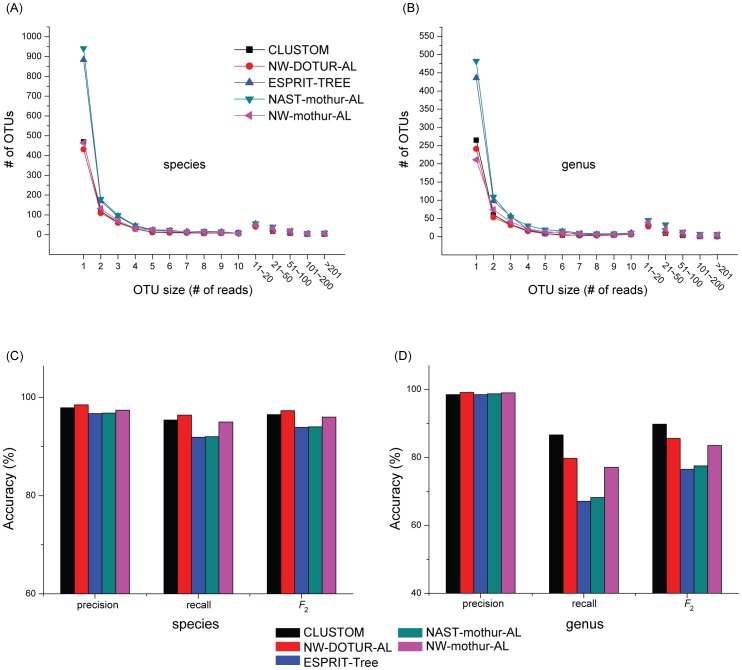
Taxonomy-based accuracy evaluation. The clustering results of CLUSTOM, NW-DOTUR-AL, ESPRIT-Tree, NAST-mothur-AL and NW-mothur-AL were evaluated using the 16S–SILVA sequences. (A–B): The number of clustered OTUs was examined at the species (3% dissimilarity; A) and genus (5%; B) levels for every OTU size (number of sequences in a cluster). (C–D): The precision and recall statistics as well as their harmonic mean (*F_2_*) [25] were used to evaluate clustering results of the five programs at the two taxonomic levels.

In order to compare the clustering accuracy between programs, we ran CLUSTOM, NW-DOTUR-AL, ESPRIT-Tree, NAST-mothur-AL and NW-mothur-AL. The PSA calculation for NW-DOTUR-AL and NW-mothur-AL was conducted according to the conditions described in *Gap treatment and NW distance* of Materials and Methods. In case of NAST-mothur-AL, the multiple sequence alignment was executed on PyNAST ver. 1.1 [27] using Greengenes ribosomal database core set as template and genetic distance was processed from mothur package by ignoring all gaps. For both DOTUR and mothur, we used the average linkage algorithm to cluster sequences. CLUSTOM and ESPRIT-Tree, both of which are AL-based programs, were run with default parameters. Under these conditions, we compared the five programs, clustering 27,213 sequences from the 16S–SILVA dataset at the levels of species (3% threshold) and genus (5%). Comparable numbers of OTUs were produced by CLUSTOM (812 OTUs at 3%; 448 at 5%), NW-DOTUR-AL (754 at 3%; 414 at 5%) and NW-mothur-AL (902 at 3%; 480 at 5%). In turn, ESPRIT-Tree (1375 at 3%; 722 at 5%) and NAST-mothur-AL (1501 at 3%; 851 at 5%) generated more OTUs than the other programs. This is an interesting result since all programs are based on the AL method and are expected to generate similar OTU numbers. To explore the cause of this difference, we further examined the number of OTUs per OTU size that illustrates the number of sequences in a cluster (Figure 4A,B). The five AL-based programs produced similar numbers of large-sized OTUs. However, ESPRIT-Tree and NAST-mothur-AL produced higher numbers of small-sized OTUs than CLUSTOM, NW-DOTUR-AL and NW-mothur-AL at both taxonomic levels, especially with numbers of reads <4. With respect to both total numbers of OTUs and their distribution along OTU sizes, CLUSTOM, NW-DOTUR-AL and NW-mothur-AL indeed behaved in a similar way but the behavior of the other programs were heterogeneous (Figure 4A,B). Since the number of OTUs does not inform us of how individual sequences are clustered, this statistics is not sufficient to compare the clustering accuracy. We thus evaluated how well the sequences of a taxon at the levels of species and genus are assigned to a single OTU with the 16S–SILVA dataset. In fact, some bacterial species share identical or almost identical 16S sequences even though they are classified into different species, even different genera, usually due to the difference of pathogenicity or host range (e.g., between *Escherichia coli and Shigella* spp., and between *Brucella* spp.) [28]. This problem appears frequently since over 670 prokaryotic species have been classified into approximately 250 species groups so far [28]. Indeed, it is impossible to separate the 16S sequences within a species group based on sequence dissimilarity. For these instances, the multiple species that belong to a species group are regarded as a composite taxon. The application of these criteria collapsed 1,138 species of 16S–SILVA into 445, 431, 546, 562, 480 and 618 taxa when clustering with CLUSTOM, NW-DOTUR-AL, and ESPRIT-Tree, NAST-mothur-AL and NW-mothur-AL, respectively. The same criteria were applied to genus level. Analysis of 236 genera of the 16S–SILVA dataset in CLUSTOM, NW-DOTUR-AL, ESPRIT-Tree, NAST-mothur-AL and NW-mothur-AL generated 166, 168, 194, 196, 176 and 205 taxa for genera. In general, tens of thousands of 16S sequences in a microbial community produce a defined number of clusters and result in the large TN values for individual taxa. In this case, the specificity value that is calculated by TN divided by the sum of TN and FP almost reaches one, regardless of the accuracy of the program tested. For this reason, we do not use the statistics of sensitivity and specificity. Another alternative is the normalized mutual information (NMI) score that has been widely used to evaluate clustering algorithms [12]. However, this score is also problematic since it penalizes the instance where sequences belonging to a species group are assigned to the same cluster. We thus computed ‘precision’ TP/(TP+FP) and ‘recall’ TP/(TP+FN) for every taxon at species and genus levels (Figure 4C,D). In terms of precision that evaluates the fraction of correctly assigned reads for individual OTUs, the accuracy of the five AL-based programs at species and genus levels was similar to each other but NW-DOTUR-AL and CLUSTOM were ranked 1^st^ and 2^nd^, respectively. On the other hand, the recall values of CLUSTOM, NW-DOTUR-AL and NW-mothur-AL were higher than ESPRIT-Tree and NAST-mothur-AL at the species level, indicating that these programs assign sequences more accurately across OTUs determined. It is noteworthy that the recall values of the five programs at the genus level are relatively lower than those at the species level (Figure 4C,D). By examining OTU distribution at the genus level, we identified that this unexpected result was caused by a couple of large-sized taxa. For example, the genus *Bacillus* is the largest in the 16S–SILVA (19% of 27,213 sequences). While CLUSTOM clustered the majority of the *Bacillus* sequences into a single OTU, they were more evenly distributed into many OTUs in the other programs.

### Distance-based Reliability Test

**Figure 5:**
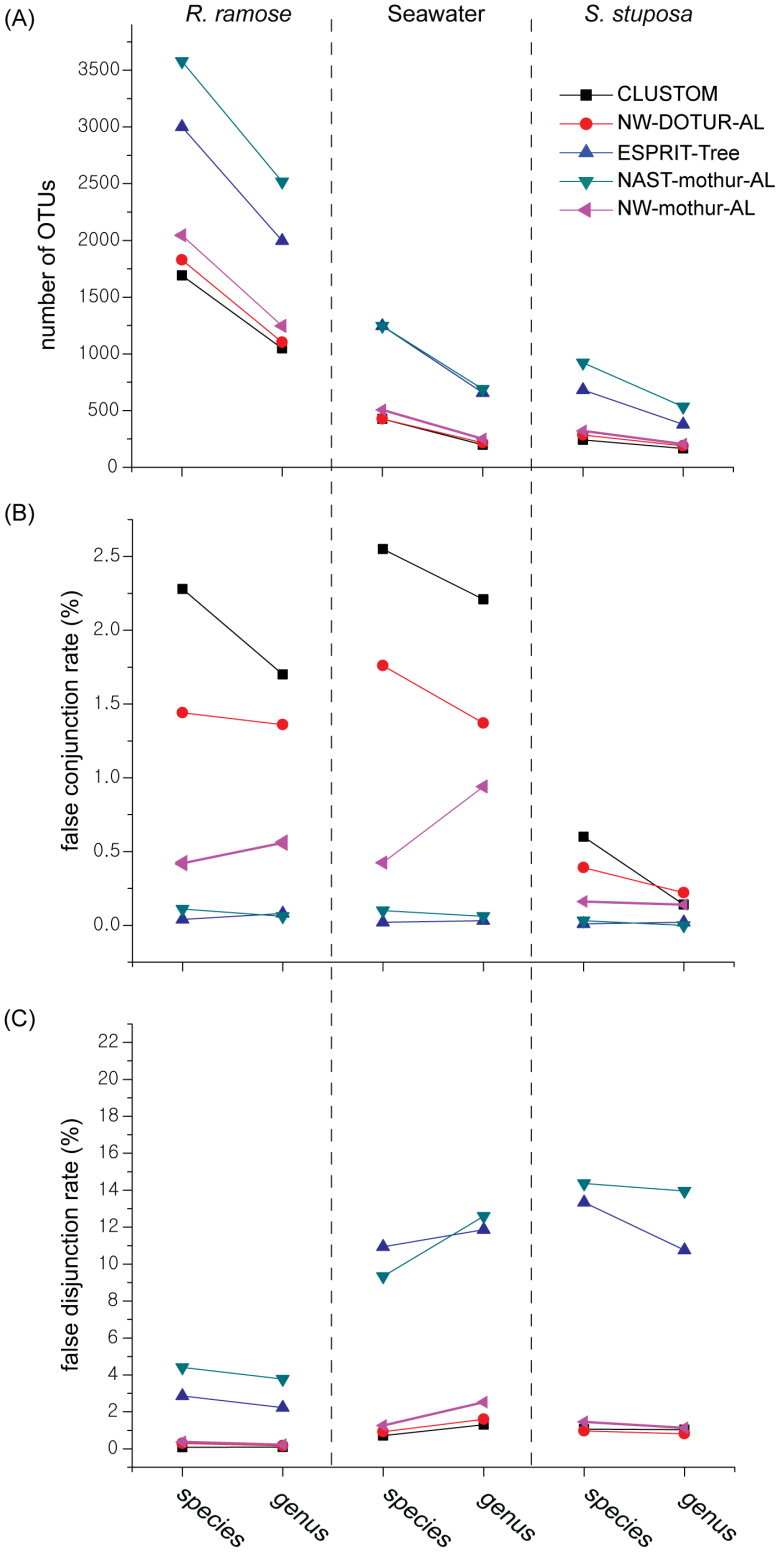
Distance-based accuracy evaluation. We ran CLUSTOM, NW-DOTUR-AL, ESPRIT-Tree, NAST-mothur-AL and NW-mothur-AL using the *R. ramose* (complex community), Seawater (intermediate), and *S. stuposa* (simple) datasets of 454–SPONGE at the species (16S distance of 3%) and genus (5%) levels. The clustering accuracy of the five programs was evaluated for each of dataset and taxonomic level with the following statistics: (A) total numbers of OTUs; (B) false conjunction rates; and (C) false disjunction rates. The definition and formulas of the false rates are described in MATERIALS AND METHODS.

For each of the three 454–SPONGE datasets, we clustered the sequences at the levels of species (3% dissimilarity) and genus (5%) using the five programs under the same settings defined above. In fact, the *R. ramose* dataset has the largest richness and its evenness is similar to the seawater dataset. In contrast, the *S. stuposa* dataset has the smallest richness and evenness values [22]. While the numbers of OTUs produced by CLUSTOM were very similar to those by NW-DOTUR-AL and NW-mothur-AL, the other programs generated many more OTUs regardless of the data structures of query samples (Figure 5A). We then tested how well individual programs clusters sequences for given distance thresholds since there was no taxonomy information available for these 454 datasets. At the levels of species and genus, rates of false conjunction and false disjunction of CLUSTOM, NW-DOTUR-AL and NW-mothur-AL were similar to each other (Figure 5B, C). On the other hand, the false conjunction rates of ESPRIT-Tree and NAST-mothur-AL were significantly lower than those of the other programs. In contrast, the false disjunction rates of ESPRIT-Tree and NAST-mothur-AL were higher than those of the other programs, revealing that the two statistics have indeed a tradeoff relationship.

### Running Time

**Figure 6:**
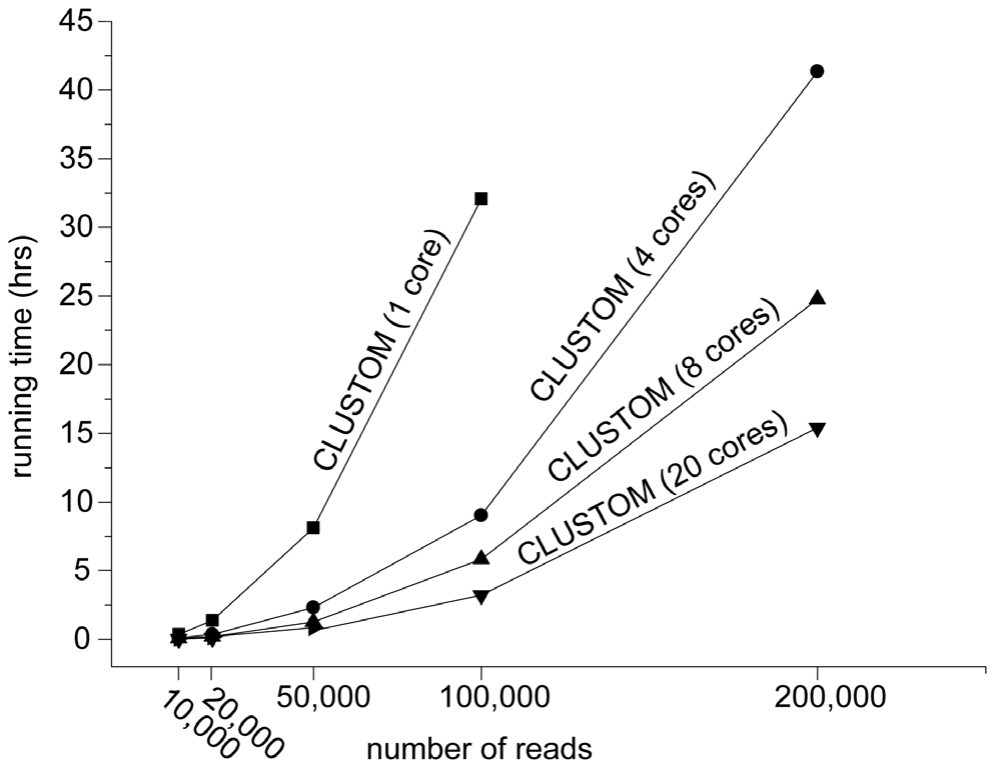
Efficiency test. The running times of CLUSTOM were measured using the 454–HMP dataset with a varying number of sequences (10 K, 20 K, 50 K, 100 K, and 200 K). The CLUSTOM algorithm exhibits a polynomial computational complexity of *O(N^2^)* when calculating *k*-mer distances between every pair of input sequences.

We assessed the running time of CLUSTOM by analyzing the four randomly sampled 454–HMP datasets, each of which had 10 K, 20 K, 100 K, and 200 K sequences. The NW calculation of NW-DOTUR-AL and NW-mothur-AL requires polynomial complexity, *O(N^2^)*, with *N* representing the number of sequences. The analysis of 10 K sequences using 20 CPU cores required ∼5 hours for NW-DOTUR-AL and NW-mothur-AL, indicating that these programs cannot handle large-scale datasets (data not shown in Figure 6). While CLUSTOM, NW-DOTUR-AL and NW-mothur-AL share the same computational complexity under the big *O* concept, CLUSTOM allots this complexity for the calculation of *k*-mer distances between sequence pairs, which can be computed much faster than NW distances [7], [8]. The analysis of the 10 K dataset under the same environment (20 cores) showed that CLUSTOM was over 150 times faster than NW-DOTUR-AL and NW-mothur-AL (0.03 versus ca. 5 hours). In addition, the 100 K dataset was processed in less than 10 hours using CLUSTOM with 4 CPU cores (Figure 6). This represents the environment of most personal computers. Although the *refinement* step requires NW distance calculation between seed sequences, this running time can be disregarded since the computation is applied to a minority of input sequences (0.1 to 4% for the datasets analyzed in this study). For the 10 K, 20 K, and 100 K datasets, the CLUSTOM run time on 20 CPU cores was comparable to that of ESPRIT-Tree that has the computational complexity of quasilinear *O(N^1.17^)* [11]. Since the calculation of NAST-based alignment can be parallelized, we did not measure the run time of NAST-mothur-AL.

## Discussion

We implemented CLUSTOM, a program that uses a new clustering algorithm capable of analyzing large 16S sequence datasets with relatively high accuracy and in a personal computer. The clustering of sequences represents the essential step in the analysis of large-scale microbial community data derived from the NGS platforms. Many programs have been developed with this goal in mind, some of which are widely used by microbiologists. Since a specific genetic distance must be defined as threshold prior to clustering, microbial researchers established the 3% and 5% thresholds to separate taxa at species and genus-level OTUs, respectively [7], [24]. However, many prokaryotic species are highly homogeneous while others harbor considerable genetic variation. Consequently, a universal threshold cannot satisfy the appropriate designation of every taxonomic unit. More importantly, the definitions of which genetic distances are the most appropriate to delimit boundaries of species and higher taxonomic units remain highly contentious [24], [29]. Consequently, any advance in our ability to cluster 16S sequences more accurately and with premises that are more realistic represents a valuable accomplishment.

The clustering accuracy largely depends on how accurately the genetic distance is measured between sequences. Recent benchmark studies revealed that the distance is more accurately calculated from a PSA than from a MSA and that AL-based hierarchical clustering methods outperform other clustering methods in terms of accuracy [11], [12], [18], [19]. Consequently AL-based NW-DOTUR-AL and NW-mothur-AL can be regarded as the ideal clustering methods. We here tested the performance of the two programs using the same datasets of NW distances. Since mothur is the recent modification of DOTUR for memory efficiency [9], the clustering results of the two programs were not significantly different (Figures 4,5). Their clustering accuracy was higher than NAST-mothur-AL, confirming the results of previous studies that NW followed by AL-based clustering is superior to other hierarchical or heuristic clustering methods (Figure 4,5). However, both programs require polynomial computational complexity in calculating NW distances. Running time limitations makes its use impossible with large-sized sequence datasets. Since many researchers select a clustering method based on speed and ease of use, ESPRIT-Tree and NAST-mothur-AL can still be good alternatives. However, speed and accuracy follow a tradeoff relationship as shown with the relatively lower accuracy of the two programs in our analysis (Figures 4,5). In fact, ESPRIT-Tree uses the BIRCH algorithm to quickly partition the space of input sequences at pre-defined distance levels and subsequently refines clusters by finding the closest pairs of sequences iteratively [8]. Any sequences within a cluster of ESPRIT-Tree should satisfy the triangular inequality of genetic distances with the center sequence of the cluster. Due to this mathematical inequality, the distance of every sequence pair within a cluster is theoretically lesser than the user-defined clustering threshold. On the other hand, ESPRIT-Tree never allows the overlap between clusters determined initially. Consequently, the program produces many free-lancer sequences in areas of sequence space that are not occupied by clusters. In case of UCLUST that is not considered in our analysis, an input sequence is either assigned to an existing cluster or regarded as a new cluster seed depending on if the genetic distance of the input sequence with the seeds of existing clusters is smaller than a user-defined threshold [5]. Consequently, the principle of partitioning the input sequence space is nearly identical between ESPRIT-Tree and UCLUST, indicating that the two programs will perform in a similar way. To fill the gaps that are produced by non-overlap initial clusters, ESPRIT-Tree produces a number of small-sized clusters (Figures 4A,B). Consequently, an input sequence space is divided into sub-spaces of various sizes by the clusters determined. This partitioning eventually causes lots of overlaps between adjacent clusters under a given distance threshold as shown by higher false disjunction rates of ESPRIT-Tree than the other programs (Figure 5C). Although NAST-mothur-AL shows accuracy similar to ESPRIT-Tree that is based on PSA, its relatively lower clustering accuracy is caused by the limitation of MSA. The multiple sequence alignment of NAST-mothur-AL was determined by running NAST program. The accuracy of this aligner largely depends on the completeness of a reference database. However, all of the current 16S databases suffer from the limited sequence information that only represents a minority of modern prokaryotes. Consequently, some of the input sequences are not comparable to sequences in the reference databases and will form independent small-sized OTUs. Although the methodological nature of NAST-mothur-AL is highly different from that of ESPRIT-Tree, the incompleteness of extant reference databases makes the clustering results of NAST-mothur-AL similar to that of ESPRIT-Tree. The two programs that have linear or quasilinear computational complexity are much faster than NW-DOTUR-AL and NW-mothur-AL. However, the large production of small-sized OTUs is indeed a serious problem. These small-sized clusters lead to overestimation of the number of rare phylotypes, which significantly influences the calculation of α–diversity indexes (e.g., ACE, Chao1, Good’s coverage) that are essential to understand the structure of microbial communities.

To our knowledge, the algorithmic implementations of existing programs developed so far are more biased towards informatic considerations. In contrast, CLUSTOM’s algorithmic implementation considers the nature of prokaryotic genetic divergence. Our study reveals the uneven distribution of 16S sequences, confirming that sequences of a species are cohesively clustered and are separated from those of other species (Figure 2). Compared to ESPRIT-Tree and NAST-mothur-AL, the distribution of OTU numbers determined by CLUSTOM was similar to those determined by NW-DOTUR-AL and NW-mothur-AL, revealing that this algorithm does not overestimate the α-diversity of a microbial community that usually results from a number of small-sized OTUs (Figures 4A, 5A). Furthermore, the lower false disjunction rates of CLUSTOM, which are comparable to those of NW-DOTUR-AL and NW-mothur-AL, reveal that our algorithm reduces the degree of overlap between adjacent clusters in comparison to ESPRIT-Tree and NAST-mothur-AL (Figure 5C). Although CLUSTOM has the highest false conjunction rates than the other four programs, the rates were only 2 to 2.5% higher than those from ESPRIT-Tree, which had the lowest false conjunction rates (Figure 5B). On the other hand, the false disjunction rates of CLUSTOM were much lower than those of ESPRIT-Tree and NAST-mothur-AL (2.5 to 15% in difference; Figure 5C). Consequently, the ability of CLUSTOM to separate 16S sequences between adjacent clusters overwhelms the shortcoming of assigning few wrong sequences into clusters, which was also supported by the taxonomy-based reliability test (Figure 4). In summary, the accuracy of CLUSTOM is higher than ESPRIT-Tree, NAST-mothur-AL and NW-mothur-AL, and is similar to that of NW-DOTUR-AL at both species and genus levels (Figures 4, 5). Since genetic homogenization decreases by lack of homologous recombination at higher taxonomic levels, we initially expected that the algorithmic premise of CLUSTOM be only applicable to sequence clustering at the species level. Remarkably and against expectations, the accuracy of CLUSTOM was better than any other programs at the genus-level, indicating that assumptions in CLUSTOM work well at both species and genus levels (Figures 4C,D). Since most microbial community studies are conducted at these taxonomical levels, our results guarantee the future successful application of the CLUSTOM implementation. Furthermore, the use of *k*-mer thresholds makes CLUSTOM much faster than NW-DOTUR-AL and NW-mothur-AL. In practice, tens of thousands of 16S sequences are sufficient for understanding a complex microbial community structure without bias. To analyze this amount of data, CLUSTOM requires less than 10 hours of run time on four CPU cores, indicating that users can handle the large-scale data on their personal computer environment (Figure 6). A parallel processing web application of CLUSTOM that processes a single query with 20 CPU cores is available to the public. This web service provides the opportunity to analyze hundreds of thousands of 16S sequences in reasonable time (Figure 6).
