# Correction: CLUSTOM: A Novel Method for Clustering 16S rRNA Next Generation Sequences by Overlap Minimization

**DOI:** 10.1371/annotation/bb3baaad-6b58-41a0-96cc-7bdc819de411

**Published:** 2013-05-06

**Authors:** Kyuin Hwang, Jeongsu Oh, Tae-Kyung Kim, Byung Kwon Kim, Dong Su Yu, Bo Kyeng Hou, Gustavo Caetano-Anollés, Soon Gyu Hong, Kyung Mo Kim

Author Soon Gyu Hong should have been noted as a Corresponding Author, with the email address polypore@kopri.re.kr

